# The Influence of Tobacco Smoke on Protein and Metal Levels in the Serum of Women during Pregnancy

**DOI:** 10.1371/journal.pone.0161342

**Published:** 2016-08-22

**Authors:** Marta Wrześniak, Marta Kepinska, Małgorzata Królik, Halina Milnerowicz

**Affiliations:** 1 Department of Biomedical and Environmental Analysis, Faculty of Pharmacy, Wroclaw Medical University, Wroclaw, Poland; 2 Early Pregnancy Pathology Clinic, Centre of Gynaecology, Obstetrics and Neonatology, Opole, Poland; Hokkaido Daigaku, JAPAN

## Abstract

**Background:**

Tobacco smoking by pregnant women has a negative effect on fetal development and increases pregnancy risk by changing the oxidative balance and microelements level. Smoking affects the concentration, structure and function of proteins, potentially leading to various negative effects on pregnancy outcomes.

**Methodology/Principal Findings:**

The influence of tobacco smoke on key protein fractions in smoking and non-smoking healthy pregnant women was determined by capillary electrophoresis (CE). Concentrations of the proteins α1-antitrypsin, α1-acid glycoprotein, α2-macroglobulin and transferrin were determined by ELISA tests. Total protein concentration was measured by the Biuret method. Smoking status was established by cotinine levels. Cadmium (Cd) and Zinc (Zn) concentrations were determined by flame atomic absorption spectrometry and the Zn/Cd ratio was calculated based on these numbers. Smoking women had a 3.7 times higher level of Cd than non-smoking women. Zn levels decreased during pregnancy for all women. The Zn/Cd ratio was three times lower in smoking women. The differences between the changes in the protein profile for smoking and non-smoking women were noted. Regarding proteins, α1-antitrypsin and α2-macroglobulin levels were lower in the non-smoking group than in the smoking group and correlated with Cd levels (r = -0.968, p = 0.032 for non-smokers; r = −0.835, p = 0.019 for smokers). Zn/Cd ratios correlated negatively with α1-, α2- and β-globulins.

**Conclusions/Significance:**

Exposure to tobacco smoke increases the concentration of Cd in the blood of pregnant women and may lead to an elevated risk of pregnancy disorders. During pregnancy alter concentrations of some proteins. The correlation of Cd with proteins suggests that it is one of the causes of protein aberrations.

## 1. Introduction

Tobacco smoke is one of the most commonly used stimulants around the world. Exposure to tobacco smoke leads to a range of negative effects in the human body and is especially dangerous for pregnant women. Tobacco smoke increases the risk of spontaneous abortion, ectopic pregnancy, intrauterine growth restriction (IUGR), fetal low birth weight (LBW) and preterm birth. Fetal disorders induced by tobacco smoke may be caused by one or more of its various components: carbon monoxide, nicotine, and heavy metals like cadmium (Cd), arsenic and lead [1]. This stimulant may influence microelement levels [2], disrupt oxidative balance [3, 4] and disturb protein concentration [5].

Tobacco smoke influences the protein profile of the serum in non-pregnant women; however, the additive effects of pregnancy and tobacco smoking on proteins have not been fully examined [6]. Prior studies have shown that alterations observed in each protein fraction are a useful indicator of the progress of diseases. In addition, changes in protein metabolism and differences in the protein structure are connected with pregnancy disorders like preeclampsia, IUGR and LBW [7].

During pregnancy, protein fraction distributions shift, with α1-, α2- and β-globulins rising and albumin and γ-globulin declining [8]. These physiological alterations are reflections of the female body’s preparation to support a proper pregnancy outcome. Any improper change in protein production may be a signal of a potentially elevated risk for the fetus and mother and could require increased monitoring of pregnant women’s health [9]. It is changes in the concentration of one or several major proteins within a particular fraction that are usually responsible for the observed changes in the level of that fraction. For the albumin fraction, the main protein is albumin; for the α1-globulin, it is mainly α1-antitrypsin and α1-acid glycoprotein; for α2-globulin, changes are found in α2-macroglobulin, haptoglobin and ceruloplasmin. The β-globulin fraction consists primarily of transferrin, β-lipoprotein and the complementary component 3, while in the γ-globulin fraction, there are γ-globulin proteins.

Improper changes in albumin concentrations can influence the balance between prooxidants and antioxidants and, as a consequence, lead to higher oxidative stress, which is associated with IUGR and other pregnancy disorders [10]. Decreased albumin levels are additionally linked to a higher risk of stroke and coronary heart disease, and may be a marker of inflammation, which is very dangerous to the mother and her baby. Similar to albumin, α1-antitrypsin is an acute-phase protein with protease inhibitor activity belonging to the α1-globulin fraction. High levels of this protein are observed in the blood of pregnant women with hypertension; however, attempts to link α1-antitrypsin with preeclampsia have proven inconclusive [11]. α1-acid glycoprotein is another protein belonging to the α1-globulin fraction. This glycoprotein is an acute-phase protein and during inflammation, its level rises. Throughout pregnancy its concentration does not change; however, changes in the glycosylation of the α1-acid glycoprotein have been observed in both disease and physiological states like pregnancy[12]. α2-macroglobulin, a glycoprotein present in the α2-globulin fraction, is considered to be an acute-phase protein [13]. It is a major endoprotease inhibitor that can stimulate diverse immune functions, as well as transport zinc (Zn) and other metals. Elevated levels of this protein may be a reflection of higher demand for Zn or changes in coagulation properties. Moreover, the α2-macroglobulin concentration correlates with infant birth weight [14]. Finally, transferrin is a protein responsible for iron transport. During pregnancy, its level rises in response to an increased demand for iron. Correlations have been noted between infant birth weight, serum transferrin concentration and its sialylation in some disorders (e.g. severe preeclampsia), but not in healthy pregnancies [15, 16].

Tobacco smoke may influence the protein concentration and metabolism through the many xenobiotics contained therein. It also acts as a carrier of Cd and is the main non-occupational source of this heavy metal. The Cd concentration in one cigarette ranges from 0.5 to 3.5 μg/g, and smoking 40 cigarettes per day provides an amount of this metal twice larger than that contained in food [17]. Cd is nephrotoxic and carcinogenic. It accumulates in the placenta and interferes with the transport of many micronutrients to the fetus. As the placenta regulates maternal and fetal transport of nutrients, the elimination of waste products, as well as the exchange of gases, any dysfunction in this organ may contribute to impaired fetal growth [18, 19] and, consequently, lower fetal birth weight [20]. Cd concentration also correlates negatively with Zn, a metal necessary for proper fetal growth. Levels of Zn are found to be much lower in smoking than in non-smoking pregnant women [21], and not just in the mother’s blood, but in the umbilical cord blood, as well. The Zn/Cd ratio is also correlated with fetal birth weight [22, 23].

The aim of the present study was to examine the influence of tobacco smoke on selected serum proteins found in the blood of pregnant women. In our study, we selected five proteins (albumin, α1-antitrypsin, α1-acid glycoprotein, α2-macroglobulin and transferrin) for which changes in concentration or structure are connected to abnormalities during pregnancy, and correlated them with tobacco smoking as a factor that has a negative impact on fetal development. We investigated if the Cd contained in tobacco smoke is associated with alterations in the concentration of the previous five proteins and whether smoking influenced the protein profile in the blood of pregnant women.

## 2. Material and Methods

### 2.1. Subjects

Healthy pregnant women (*n* = 55) with proper pregnancy outcomes were admitted to the study in the years 2013 to 2015. Blood was collected from pregnant women in each trimester of pregnancy. Some patients joined during a later term of pregnancy, and four left before delivery (though information about the infant and delivery was still collected). The gestational age was determined by the date of the last menstrual period and confirmed by ultrasound examination. Samples were divided into two subgroups: S for smoking (24 samples) and NS for non-smoking (78 samples) as a control group.

Blood samples were collected in the 1^st^ trimester (up to 12 weeks in gestational age), 2^nd^ (from 13 to 26 weeks in gestational age) and 3^rd^ (27 or more weeks in gestational age) during regularly scheduled appointments with a physician at a gynecological clinic in Poland. Venous blood samples were obtained following the standard procedure, using tubes with heparin for plasma (S-Monovette, Sarstedt, No: 04.1907) and with a clotting activator (S-Monovette, Sarstedt, No: 04.1905) for serum preparation. Serum and plasma samples were centrifuged at 2500xg for 17 min to separate the serum from the plasma. Specimens were immediately frozen (-70°C) until further use.

### 2.2. Exclusion criteria

Pregnant women with diagnosed IUGR, diabetes, insulin resistance, or thyroid disease and those whose pregnancy ended in miscarriage were excluded. Fetuses with diagnosed infections, malformations and genetic aberrations disqualified women from the study, as well. Data on the number of selected pregnant women, rejections and exclusions are included in the supplementary material: S1 and S2 Tables.

### 2.3. Ethical clearance

Pregnant women were approved for the study by the Local Bioethics Committee of Wroclaw Medical University (KB– 845/2012). Participants provided written consent to participate in this study. The Local Bioethics Committee of Wroclaw Medical University approved this consent procedure.

### 2.4. Smoking status, Cd and Zn concentrations, and Zn/Cd ratio

Smoking status was established by measuring the concentrations of the nicotine metabolite cotinine, using the commercial, indirect immunoenzymatic method (Cotinine, LUCIO-Direct ELISA, No: 501.301). According to the manufacturer’s instructions, all samples with cotinine levels above 25 ng/ml were admitted to the smoking group.

Cd concentration was determined in the blood samples by graphite furnace atomic absorption using SOLAAR M6, Thermo Elemental Co., at 228.8 nm wavelength with the Zeeman background correction. The Zn concentration in plasma was determined by flame atomic absorption spectrometry, with absorbance measurements taken at 213.9 nm wavelength in acetylene and air flame, with a deuterium background correction. Reference materials (Recipe, BCR) were used to determine the calibration curve and controls. The Zn/Cd ratio was calculated by dividing the Zn concentration [μg/l] by the Cd concentration [μg/l].

### 2.5. Determination of total protein concentration

Total protein concentration was determined by the Biuret method with copper (II) sulfate in an alkaline solution. In brief, when in the presence of peptide bonds, the Biuret reagent (sodium hydroxide / hydrated copper[II] sulfate and potassium sodium tartrate) forms violet-colored coordination complexes which are detected at 540 nm. The intensity of the color is directly proportional to the protein concentration [24].

### 2.6. Serum protein separation

Separation of the serum proteins was performed on the Beckman Coulter PA800plus capillary electrophoresis (CE) system, Pharmaceutical Analysis System. A fused-silica capillary of 25 μm internal diameter and 30 cm length from the Beckman Coulter and CEofix SPE kit (No: P1310-004188) for Beckman Coulter from Analis (No: P10-004750) was used. The analysis was carried out according to the manufacturer's instructions, with 10 cm to the separation window (as an effective length) used. After installation of the capillary, conditioning (with 0.2 M NaOH) was performed.

Before each run, the capillary was coated by an initiator, a Tris buffer of pH 9.7, at 25 psi for half a minute. Next, the capillary was rinsed by a separation buffer of Tris/taurine of pH 9.7 at 25 psi for 1.5 min. Serum samples were injected using 0.5 psi for 4 sec. Separation was carried out by applying 15.0 kV for 5 min with the inlet as the cathode and the outlet as the anode. Each run ended with rinsing the capillary with a conditioner.

Serum protein separation was conducted inside the capillary under an electrical field. Proteins with a negative charge, which were electrically attracted to the anode, were progressively retarded in accordance with their charge/mass ratio. Protein presence was detected at 214 nm.

Karat32 ver. 9.0 software (Beckman Coulter Inc., Brea, CA, USA) was the operating system used to acquire and analyze data. To analyze the integration parameters, the instructions for the ‘CEofix SPE kit for Beckman Coulter P/ACE MDQ series’ test, Ref No: 10–004750, were applied. Five peaks between the time from 1 to 5 min of separation were expected. The area of each peak, representing the different protein fractions (albumin, α1-globulins, α2-globulins, β-globulins, γ-globulins), was calculated as a percentage area in relation to the total area of all detected proteins. Serum proteins level were converted to g/l of total protein.

### 2.7. Determination of α1-antitrypsin, α1-acid glycoprotein, α2-macroglobulin and transferrin

ELISA tests were used to determine α1-antitrypsin (α1-Antitrypsin Clearance ELISA, Immundiagnostik, No: K6752), α1-acid glycoprotein (Human alpha-1-Acid Glycoprotein ELISA kit, AssayPro No: EG5001-1), α2-macroglobulin (α2-macroglobulin ELISA Kit, Immundiagnostik, No: K6610A) and transferrin (AssayMax Human Transferrin ELISA Kit, AssayPro, No: ET2105-1) levels. Prior to use, each sample was diluted (1:40000 for α1-antitrypsin, 1:1000 for α1-acid glycoprotein, 1:50000 for α2-macroglobulin and 1:20000 for transferrin test) with a proper diluent as per manufacturer suggestions. Absorbance measurement was performed on Multiskan Go (Thermo Scientific) equipment. In order to monitor the analytical accuracy of measurements, serum controls were used in each test.

### 2.8. Statistical methods

Normality of the distribution was tested by the Shapiro-Wilk test and the equality of variances by Levene’s test. When distribution and variances were normal, ANOVA testing was used. In the event of a lack of a normal distribution and variance uniformity, the differences between groups were analyzed by the Kruskal-Wallis test. Correlations were described as Spearman's rank correlation coefficient (r). In all analyses, p<0.05 was considered statistically significant. Statistical analyses were conducted using Statistica Software Package, version 10 (Polish version; StatSoft, Poland).

## 3. Results

### 3.1. Clinical characteristics

Clinical characteristic groups are shown in Table 1. Patient groups were statistically similar in terms of maternal age, BMI before pregnancy and infant birth weight.

**Table 1:** Clinical characteristics of and blood metal concentrations in pregnant women.

Trimesters of pregnancy	Groups	Maternal age [year] [X±SD]	BMI before pregnancy [kg/m^2^] [X±SD]	Infant birth weight [g] [X±SD]	Cotinine [ng/ml] [X±SD]
**1st trimester**	**S *n = 6***	28.86 ± 6.87	23.71 ± 2.55	3258.3 ± 255.8	80.71 ± 18.53*
	**NS *n = 18***	30.38 ± 4.07	22.85 ± 3.56	3392.1 ± 412.4	3.23 ± 1.20*
**2nd trimester**	**S *n = 7***	28.50 ± 6.92	24.78 ± 3.70	3465.6 ± 213.4	72.36 ± 20.76**
	**NS *n = 35***	28.95 ± 4.32	23.46 ± 3.30	3446.6 ± 452.7	3.26 ± 1.90**
**3rd trimester**	**S *n = 10***	28.58 ± 4.25	24.90 ± 3.81	3385.5 ± 428.8	77.28 ± 16.09***
	**NS *n = 31***	29.62 ± 4.98	23.44 ± 4.03	3430.0 ± 459.4	2.90 ± 1.04***

**Groups–**S: smoking; NS: non-smoking.

*^,^ **^,^*** significant (p < 0.00) for the same parameter in the same trimester for different smoking groups (e.g. *NS group in the 1^st^ trimester has lower level of cotinine than S group in the same trimester)

### 3.2. Cotinine, Cd, Zn concentration and Zn/Cd ratio

Cotinine levels in the blood of smoking women were nearly 24 times higher than in the blood of non-smoking women in the each trimester of pregnancy (1^st^: 80.71 ng/ml vs 3.23 ng/ml; 2^nd^: 72.36 ng/ml vs 3.26 ng/ml; 3^rd^ 77.28 ng/ml vs 2.90 ng/ml) (Table 1).

**Fig 1:**
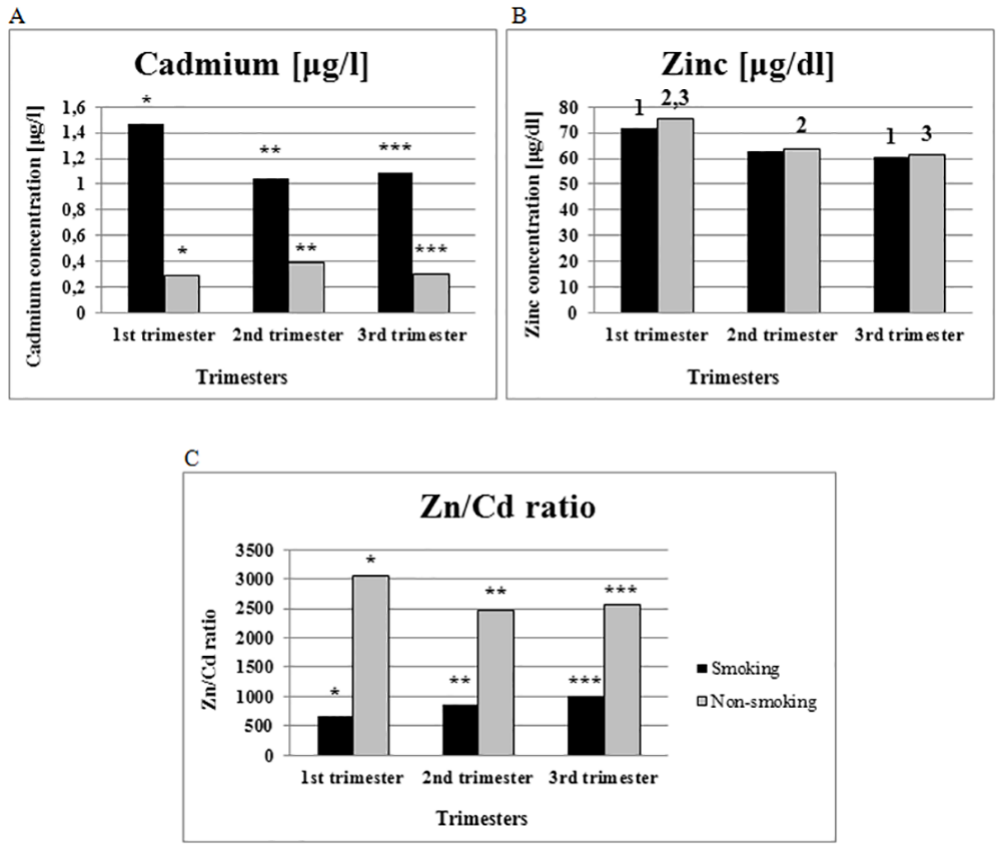
Metals concentration (A—cadmium; B—zinc) and Zn/Cd ratio (C) in smoking and non-smoking women during pregnancy. ^**1, 2,3**^ **significant** (p < 0.04) for the Zn in the same group to the other trimester (e.g. ^1^S group in the 1^st^ trimester has higher level of Zn than in the 3^rd^ trimester). *^-^*** significant (p < 0.02) for the same parameter in the same trimester for different smoking group (e.g. *S group in the 1^st^ trimester has higher level of Cd than NS group in the same trimester).

Cd concentrations in the blood of smoking women were 3.7 times higher than in non-smoking women in all trimesters: 1^st^ (1.47 μg/l vs 0.29 μg/l), 2^nd^ (1.04 μg/l vs 0.39 μg/l) and 3^rd^ (1.09 μg/l vs 0.30 μg/l) (Fig 1A).

Zn concentration declined during pregnancy in both the smoking and non-smoking group. In the blood of women in the 1^st^ trimester, there was a statistically significant higher level of Zn in both groups than in the 3^rd^ trimester (75.64 μg/dl vs. 61.68 μg/dl for non-smokers; 71.48 μg/dl vs. 60.64 μg/dl for smokers) (Fig 1B).

Zn/Cd ratios in the smoking group were significantly lower than in non-smoking group in all trimesters; in the 1^st^ trimester, smoking women recorded a Zn/Cd ratio of 663.81, while non-smoking women had 3052.43 (Fig 1C). A similar observation was made in the 2^nd^ (854.71 vs. 2461) and 3^rd^ trimesters of pregnancy (1005.70 vs. 2553.92).

### 3.3. Total protein concentration

Total protein concentration declined during pregnancy both groups (Table 2). In the 1^st^ trimester, there was a statistically significantly higher concentration of total protein than in the 3^rd^ one for both the smoking (80.16 μg/dl in the 1^st^ trimester vs 74.49 μg/dl in the 3^rd^ trimester) and non-smoking group (1^st^: 79.66 μg/dl vs. 3^rd^: 76.05 μg/dl).

**Table 2:** Concentration of α1-antitrypsin, α2-macroglobulin, α1-acid glycoprotein and total protein concentration in the serum of women during each trimester of pregnancy.

		Total protein [g/l]	α1-antitrypsin [mg/dl]	α1-acid glycoprotein [μg/dl]	α2-macroglobulin [g/l]	Transferrin [g/l]
**1st trimester**	**S *n = 6***	80.16 ± 3.70^1^	311.71 ± 40.45*	1143.02 ± 211.52	3.44 ± 0.44	3.34 ± 0.51^5^
	**NS *n = 18***	79.66 ± 4.22^2^	231.24 ± 97.17*,^3^	1078.69 ± 240.27	3.20 ± 0.57^4^	3.67 ± 0.54^6^
**2nd trimester**	**S *n = 7***	78.62 ± 4.54	289.56 ± 72.23	1139.35 ± 216.29	4.37 ± 1.14**	4.02 ± 0.24
	**NS *n = 35***	78.13 ± 5.83	290.38 ± 98.39	1058.62 ± 238.09	3.65 ± 0.94**	4.10 ± 0.59^7^
**3rd trimester**	**S *n = 10***	74.49 ± 3.37^1^	319.57 ± 95.83	1117.24 ± 212.69	3.56 ± 1.25	4.46 ±0.77^5^
	**NS *n = 31***	76.05 ± 4.17^2^	314.13 ± 110.40^3^	1092.74 ± 202.57	3.81 ± 0.64^4^	4.49 ± 0.62^6^^,^^7^

**Groups–**S: smoking; NS: non-smoking.

*^-^** significant (p < 0.02) for the same parameter in the same trimester for different smoking groups (e.g. *S group in the 1^st^ trimester has higher levels of α1-antitrypsin than NS group in the same trimester)

^**1–7**^ **significant** (p < 0.05) for the same parameter in the same group to the other trimester (e.g. ^1^S group in the 1^st^ trimester has higher levels of total protein concentration than in the 3^rd^ trimester)

### 3.4. The level of serum protein fractions

**Fig 2:**
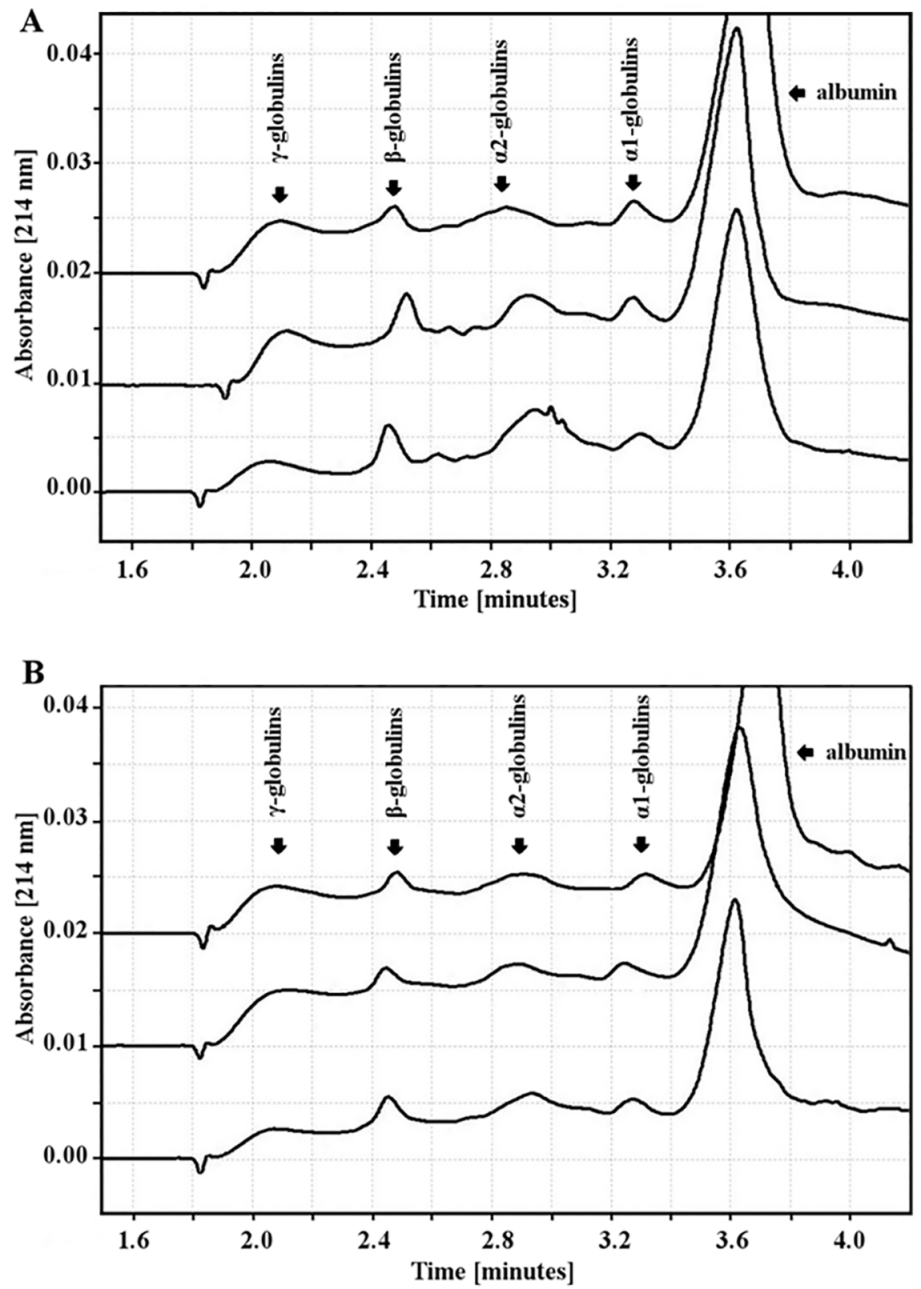
Exemplary profiles of the serum proteins of smoking (A) and non-smoking (B) women during pregnancy.

The values obtained for each protein fraction are shown in Table 3. Of note, the albumin level was statistically significantly higher in the 1^st^ trimester than in the 3^rd^ one in both smoking (50.24 g/l vs 39.53 g/l) and non-smoking women (46.55 g/l vs 42.57 g/l) (Fig 2A and 2B).

**Table 3:** Concentration of serum protein fractions in the blood of women during pregnancy.

		Albumin [g/l]	α1-globulins [g/l]	α2-globulins [g/l]	β-globulins [g/l]	γ-globulins [g/l]
**1st trimester**	**S* n = 6***	50.24 ± 1.71^1^	3.31 ± 0.75	10.88 ± 1.29^5^^,^^6^	7.44 ± 1.74	5.94 ± 1.22 **
	**NS* n = 18***	46.55 ± 4.50^2^	3.47 ± 1.20^3^^,^^4^	10.98 ± 2.28^7^^,^^8^	7.21 ± 1.72^9^^,^^10^	8.01 ± 1.70^11^^,^^12^^,^ **
**2nd trimester**	**S* n = 7***	45.89 ± 6.22	3.97 ± 0.97	12.34 ± 2.48^5^	7.36 ± 1.82	5.23 ± 1.55***
	**NS* n = 35***	44.95 ± 5.59	4.49 ± 1.38^3^	12.85 ± 2.25^7^	8.70 ± 1.36^9^	6.49 ± 1.13^11^^,^ ***
**3rd trimester**	**S* n = 10***	39.53 ± 4.31^1^	4.25 ± 1.18	15.54 ± 2.58^6^	8.06 ± 1.01*	5.61 ± 1.23
	**NS* n = 31***	42.57 ± 5.48^2^	4.94 ± 1.23^4^	14.22 ± 2.51^8^	9.57 ± 1.77^10^^,^ *	5.85 ± 1.76^12^

**Groups–**S: smoking; NS: non-smoking.

^**1–12**^ **significant** (p < 0.00) for the same parameter in the same group to the other trimester (e.g. ^1^S group in the 1^st^ trimester has higher albumin concentration than in the 2^nd^ trimester)

*, **, *** significant (p < 0.05) for the same parameter in the same trimester for different smoking groups (e.g. *S group in the 3^rd^ trimester has lower levels of β-globulins than NS group in the same trimester)

The α1-globulin concentration was lower in the 1^st^ trimester than in the 3^rd^ one in the smoking and non-smoking groups (respectively: 3.31 g/l vs. 4.25 g/l; 3.47 g/l vs. 4.94 g/l); however, only in the non-smoking group was this elevation statistically significant. The α2-globulin concentration was also statistically significantly lower in the 1^st^ trimester than in the 3^rd^ one in the blood of smoking and non-smoking women (respectively: 10.88 g/l vs 15.54 g/l; 10.98 g/l vs 14.22 g/l).

The β-globulin concentration was lower in the 1^st^ pregnancy trimester than in the 3^rd^ one in the smoking and non-smoking group (respectively: 7.44 g/l vs 8.06 g/l; 7.21 g/l vs 9.57 g/l), but it was statistically significant only for the non-smoking group. In the 3^rd^ trimester of pregnancy, smoking women had statistically significant lower levels of β-globulins than non-smoking women.

There was a higher concentration of γ-globulins in the 1^st^ than in the 3^rd^ trimester of pregnancy in the non-smoking group (8.01 g/l vs 5.85 g/l). In the smoking group, however, γ-globulins in the 1^st^ and 3^rd^ trimesters were at similar concentrations (5.94 g/l vs 5.61 g/l). There were statistically significant differences between the smoking and non-smoking groups in the 1^st^ trimester (5.94 g/l vs 8.01 g/l) and 2^nd^ trimester (5.23 g/l vs 6.49 g/l) in the concentration of γ-globulins.

### 3.5. α1-antitrypsin, α1-acid glycoprotein, α2-macroglobulin and transferrin concentration

α1-antitrypsin concentrations were lower in the 1^st^ trimester than in the 3^rd^ one in the non-smoking group (231.24 mg/dl vs. 314.13 mg/dl). In the smoking group, this change was not observed. In the 1^st^ trimester, α1-antitrypsin levels were higher in the blood of smoking than non-smoking women.

The concentration of α1-acid glycoprotein did not change during pregnancy and was at a similar level in the blood of both smoking and non-smoking women (Table 2).

The concentration of α2-macroglobulin was lower in the 1^st^ trimester than in the 3^rd^ one in the non-smoking group (respectively: 3.20 g/l vs 3.81 g/l). In the 2^nd^ trimester, α2-macroglobulin levels were higher in the blood of smoking than non-smoking women.

Transferrin concentration was lower in the 1^st^ trimester than in the 3^rd^ one in both groups (3.34 g/l vs 4.46 g/l for smokers; 3.67 g/l vs 4.49 g/l, for non-smokers).

### 3.6. Correlations

Correlations between the cotinine, Cd and Zn levels as well as the Zn/Cd ratio, and the concentration of proteins for the smoking group are shown in Table 4.

**Table 4:** Correlations between the concentrations of total protein, serum proteins, α1-antitrypsin, α1-acid glycoprotein, α2-macroglobulin, transferrin, with the Zn/Cd ratio and cotinine, Cd, and Zn concentrations in pregnant smoking women [n = 10] in the 3rd trimester of pregnancy.

	Cotinine [ng/ml]	Cadmium [μg/l]	Zinc [μg/l]	Zn/Cd ratio
**Total protein** **[g/l]**	n/s	r = −0.903 p = 0.002	n/s	n/s
**Albumin** **[g/l]**	r = −0.788 p = 0.020	n/s	n/s	n/s
**α1-globulins** **[g/l]**	r = 0.840 p = 0.002	r = 0.672 p = 0.035	n/s	r = −0.763 p = 0.046
**α2-globulins** **[g/l]**	r = 0.741 p = 0.035	r = 0.787 p = 0.021	n/s	r = −0.764 p = 0.046
**β-globulins** **[g/l]**	n/s	n/s	r = −0.831 p = 0.041	r = −0.958 p = 0.042
**γ-globulins** **[g/l]**	n/s	n/s	n/s	n/s
**α1-antitrypsin** **[mg/dl]**	n/s	r = −0.968 p = 0.032	n/s	n/s
**α1-acid glycoprotein** **[μg/dl]**	n/s	n/s	n/s	n/s
**α2-macroglobulin** **[g/l]**	r = 0.768 p = 0.044	r = 0.835 p = 0.019	n/s	n/s
**Transferrin** **[g/l]**	r = 0.736 p = 0.024	n/s	r = −0.731 p = 0.039	n/s
**Cadmium** **[μg/l]**	r = 0.624 p = 0.040	-	r = −0.823 p = 0.023	r = −0.968 p = 0.000
**Infant Birth Weight** **[g]**	n/s	r = −0.799 p = 0.003	n/s	n/s

**n/s:** non-significant when p>0.05

Correlations (p<0.05) with cotinine concentration were observed for albumin (r = −0.788), α1-globulins (r = 0.840), α2-globulins (r = 0.741) and α2-macroglobulin (r = 0.768), transferrin (r = 0.736), and Cd (r = 0.624).

Correlations (p<0.05) with Cd concentration were obtained for total protein (r = −0.903), α1-globulins (r = 0.672), α2-globulins (r = 0.787), α1-antitrypsin (r = −0.968) and α2-macroglobulin (r = 0.835). Infant birth weight correlated (p< 0.003) with Cd as well (r = −0.799).

Zn concentrations correlated (p<0.05) with β-globulins (r = −0.831) and transferrin (r = −0.731). Zn/Cd ratios correlated (p<0.05) with α1-globulins (r = −0.763), α2-globulins (r = −0.764), β-globulins (r = −0.958) and Cd (r = −0.968).

## 4. Discussion

Around 12% of women smoke during pregnancy [25]. Tobacco smoking by pregnant women negatively influences fetal development and increases risks for pregnancy. Moreover, tobacco smoke during pregnancy has a negative effect on infants and toddlers, leading to impaired social behavior and a higher risk of childhood cancer. Tobacco smoking during pregnancy elevates the risk of sudden infant death syndrome (SIDS) with a dose-response effect exhibited between maternal smoking and risk of SIDS [1]. Tobacco smoke influences the level of protein production, degradation and modification (e.g. sialylation) [7, 26]. Exposure to tobacco smoke additionally influences the inflammation what may be seen by raised levels of C-reactive protein and white blood cells [27]. Finally, changes in the oxidative balance stimulate the body to produce proteins responsible for re-balancing [3, 28], and tobacco smoke consists of many components which may be responsible for causing an imbalance—nicotine, carbon monoxide and heavy metals. Each of them may influence protein metabolism and micronutrient levels and, in consequence, may lead to negative pregnancy outcomes.

### The impact of smoking on metal levels in the blood of pregnant women

In our study, smoking women had around 24-fold higher levels of cotinine than non-smoking women, confirming the usefulness of cotinine as a marker of current tobacco smoking. To determine long-term tobacco smoke exposure, Cd levels are a more suitable indicator [29]. Cd concentration in the blood of smoking women was 3.7-times higher than in the blood of non-smoking women, which is consistent with other research and points to tobacco smoke as a significant source of this heavy metal [3, 7, 30]. Cd concentrations in the blood correlated negatively with Zn levels (r = −0.823, p = 0.023) even if no differences between smoking and non-smoking women in the concentration of Zn were observed. During pregnancy, Zn concentrations in the women’s blood decrease; simultaneously, there is increasing demand for this element by the fetus. The placenta collects Cd, which may induce synthesis of the metallothionein, which in turn may lead to Zn being retained in the placenta instead of being transferring in proper amounts to the fetus. Insufficient Zn transport to the fetus may lead to fetal malformations [21]. In our study, the Zn/Cd ratio was between 2.5 and 3 times lower in the smoking group than in the non-smoking one across all trimesters. This disproportion may result in pregnancy disorders, as lower Zn/Cd ratios observed in the placenta have been correlated with LBW [23]. However, low placental nutrient-pollutant ratios could be considered as an additional harmful factor affecting fetal growth in mothers with a specific metallothionein genotype, one that is not present in all women [31]. Moreover, decreasing concentrations of Zn were observed with simultaneous rises in Cd levels, which may lead to impairment in the transfer of Zn to the fetus and, consequently, result in IUGR. In some studies, it was observed that mothers who delivered their baby pre-term had higher Cd concentrations in the placenta than mothers who delivered at term. In addition, cadmium exposure may lead not only to disorders during pregnancy also but to postnatal effects, such as aberrant immune function [18, 32]. Moreover, as cadmium may disturb vitamin D metabolism in the kidney, it is therefore capable of interfering with other nutrients [19].

### Tobacco smoking’s influence on protein metabolism

Changes in the protein profile were observed for both physiological and pathological processes. During pregnancy, total protein concentration in the blood of both smoking and non-smoking women decreased, with a higher average decrease for smoking women (7.07% for smokers vs. 4.53% for non-smokers). Exposure to tobacco smoke may disturb protein synthesis due to its influence on liver function [26]. In addition, Cd present in tobacco smoke damages renal function and leads to higher proteinuria [33]. The difference in total protein loss between smoking and non-smoking pregnant women may be a signal of inappropriate liver function or of accelerated protein degradation processes in smokers due to the higher Cd concentration from tobacco smoke. As changes in total protein concentration are a reflection of abnormalities in differential metabolism of different proteins, we wanted to examine a few proteins for which improper levels may have an impact on pregnancy outcomes and fetal development. As tobacco smoke and Cd have inflammatory properties, the proteins we selected characterize both inflammatory response and are important in pregnancy maintenance. Moreover, the proteins were correlated not only with cotinine level but also with Cd level, which is a known toxic component of tobacco smoke.

### Tobacco smoking’s influence on albumin

Albumin concentrations decline during pregnancy. This change may be explained by physiological increase in the level of plasma in relation to this protein [8]. In our study, this dependence occurred in the blood of both smoking and non-smoking women. Despite the fact that there was no statistically significant change between the concentration of albumin in smoking and non-smoking women, the average decrease in albumin levels during pregnancy was higher in the blood of smoking women than in the non-smoking women (21.32% vs 8.55%). In our study, albumin levels correlated negatively with cotinine level, but not with Cd, which was reflected in other research [34]. Cadmium in the blood binds to albumin as well as metallothionein. Although Cd levels did not correlate with albumin concentration, the lower level of albumin appearing simultaneously with insufficient metallothionein concentration may still lead to tubular damage, as not all Cd is captured by this protein [19].

A lowered albumin concentration, as observed in the blood of smokers, is connected with a higher risk of stroke and coronary heart disease. Serum albumin may be a marker of susceptibility to atherosclerosis. However, there have been no studies performed examining pregnant women, smoking and albumin levels in connection with risk of stroke and coronary heart disease; therefore, we can only suspect that improper albumin concentrations may be a predictor of negative pregnancy outcomes. However, it is known that atherosclerosis and impaired flows in uteroplacental spiral arteries are observed in preeclampsia [35] and may lead to IUGR [20], so it is possible that there is a connection. In addition, albumin is a negative acute-phase protein. In the case of an increased inflammatory state, albumin synthesis in the liver is switched to other acute-phase proteins [36]. An elevated inflammatory state may lead to miscarriage, preterm birth, LBW and poor pregnancy outcomes [37]. Moreover, albumin binds metal ions (like Zn) and is recognized as an antioxidant [10]. Smoking women who have lower levels of this protein in the blood may not be protected from the influence of Cu (II) and Fe (II) reactions with hydrogen peroxide and the formation of hydroxyl radicals, which may cause elevated oxidative stress and lead to fetal disorders. And imbalanced oxidative state may lead to IUGR [3] or preeclampsia [38]. It has been shown that mothers with a pro/antioxidant imbalance during pregnancy gave birth to infants with LBW more often [3]. This shows that decreases in albumin concentration by tobacco smoke may influence fetal development in different ways.

### Tobacco smoking’s influence on α1-antitrypsin

α1-antitrypsin is a protein displaying protease inhibitor activity. It is an acute phase protein with an immunoregulatory function. Inflammation processes increase the concentration of this protein [39, 40]. α1-antitrypsin belongs to the α1-globulin fraction, whose levels rise during pregnancy, but only in the non-smoking group. There are some studies showing no significant differences in the concentration of α1-antitrypsin between consecutive pregnancy trimesters [40]; however, in our study, α1-antitrypsin concentration rose across trimesters in the non-smoking group (231.24 mg/dl at the 1^st^ trimester and 314.13 mg/dl at the 3^rd^ one). Furthermore, the concentration of α1-antitrypsin was much higher in the smoking group than in the non-smoking group in the 1^st^ trimester (311.71 mg/dl vs 231.24 mg/dl), and remained at a consistently high level throughout the pregnancy. There is no information about how tobacco smoking influences α1-antitrypsin concentration during consecutive trimesters of pregnancy, though high concentrations of this protein have been observed in pregnancies of women suffering from hypertension [41]. Similar observations were made by Catarino et al., where preeclamptic women had higher values of α1-antitrypsin than normotensive pregnant women [42]. However, in the study performed by Twina et al, women with preeclampsia had lower levels of α1-antitrypsin [11]. These ambiguous results confirm that the influence of tobacco smoke and the role of α1-antitrypsin in preeclampsia development is complex [43].

In our study, α1-antitrypsin correlated negatively with Cd. This correlation is surprising, as Cd has a pro-inflammatory impact and α1-antitrypsin is a positive acute-phase protein [39]; however, these findings are not new [22]. There have been no studies on the correlations of Cd and α1-antitrypsin in the blood of smoking pregnant women. Research performed on smelters’ blood showed a negative correlation between Cd concentration and α1-antitripsin leading to emphysema [22]. It proves that pregnancy, connected with its gradual increase of α1-antitripsin, may have a protective effect on the pregnant woman’s body. Moreover, α1-globulins correlated positively with cotinine levels, but α1-antitripsin does not share this correlation. It suggests that elevated levels of α1-globulins are due to other proteins than α1-antitripsin.

### Tobacco smoking’s influence on α1-acid glycoprotein

α1-acid glycoprotein is another protein of the α1-globulin fraction which is responsible for inflammatory response [12]. In our study, the concentration of α1-acid glycoprotein did not change during pregnancy, which is consistent with other research [44]. Smoking status did not influence levels of this protein. α1-acid glycoprotein concentration was at a physiologically appropriate level, suggesting a lack of acute-phase reaction due to inflammatory response. Despite that, there were no changes in the concentration of this protein in the blood of smoking or non-smoking pregnant women; changes were instead observed in the glycosylation of α1-acid glycoprotein [12]. In a study done by Patel et al., a positive correlation between this protein and Cd concentration was recorded in the blood of rats with a dose-related response [45]. However, α1-acid glycoprotein concentration in our study was not correlated with Cd. In addition, as tobacco smoke changes the sialylation of other proteins (like transferrin) [7], it is possible that the negative influence of smoking should be looked for in differences in glycosylation, not only in the concentration of proteins.

### Tobacco smoking’s influence on α2-macroglobulin

α2-macroglobulin is a glycoprotein mainly synthesized in the liver although it can be expressed in other tissues such as the heart or reproductive tracts. It belongs to the α2-globulin fraction. In our study, the α2-globulin fraction rose during pregnancy in the blood of both smoking and non-smoking women; in contrast, α2-macroglobulin increased during pregnancy only in the non-smoking group. What is more, in the 2^nd^ trimester of pregnancy, smoking women exhibited higher concentrations of this protein in the blood than non-smoking women (4.37 g/l vs 3.65 g/l). Tobacco smoke influences α2-macroglobulin levels by increasing oxidative stress and exerting an inflammatory stimulus which activates acute-phase proteins. The α2-macroglobulin may be a predictor of LBW [14, 46]. Additionally, this protein is able to transport Cd in the blood [47]. In our study, α2-macroglobulin was positively correlated both with cotinine and Cd levels. Rising levels of α2-macroglobulin may be a reflection of the higher procoagulant properties observed in smokers [48]. Thrombosis is dangerous to both mother and fetus, leading to fetal retardation. In addition, in many studies high levels of α2-macroglobulin and high level of Cd are correlated with lower birth weights. This may be one of the ways in which exposure to tobacco smoke disturbs pregnancy outcomes [46].

### Tobacco smoking’s influence on transferrin

Transferrin is a glycoprotein involved in iron transport. It is synthesized mainly by the liver but can be expressed by Sertoli cells, as well. It is very heterogeneous with respect to a number of sialic acids residues. During pregnancy, it is one of the most important proteins as it transfers iron to the placenta [49]. Transferrin belongs to the β-globulin fraction, whose concentration rises in consecutive trimesters of pregnancy in the blood of non-smoking women. The β-globulin fraction was higher in the 3^rd^ trimester of pregnancy for the smoking group than non-smoking groups; however, there were no differences between the groups in transferrin concentration in any trimester. As this protein is an iron carrier, its concentration rises with the increased demand for oxygen as the fetus grows. In our study, transferrin concentration correlated positively with cotinine levels but not with Cd. Moreover, there was a negative correlation between transferrin and Zn observed. Transferrin levels decline when iron stores are at high levels. Elevated concentrations of iron may lead to oxidative stress. As a result, Zn levels increase as an important element of antioxidant response [28]. Furthermore, as Cd negatively correlates with Zn, smoking women with higher levels of free iron are more exposed to increased oxidative stress.

### Tobacco smoking’s influence on γ-globulins

The γ-globulin is a fraction composed mainly of immunoglobulin. During pregnancy, our study showed the γ-globulin fraction decline in the non-smoking group only. Concentrations of γ-globulins were much lower in the smoking group than in the non-smoking group in the 1^st^ (respectively: 5.94 g/l vs 8.01 g/l), and 2^nd^ trimester (respectively: 5.23 g/l vs 6.49 g/l), and remained at a constantly low level. Decreased levels of γ-globulins may contribute to elevated susceptibility of the mother to infection. The increased frequency of infections, and the possibility of their growing more severe, is dangerous for pregnancy outcomes and can lead not only to fetal growth inhibition but also to severe fetal developmental disorders, preterm birth and miscarriage. Lowered levels of γ-globulins in the mother’s blood may limit transport of immunoglobulins to the fetus and, in consequence, damage newborns’ immune defense. Moreover, infants whose mothers smoked during pregnancy and continued to do so after delivery had a higher risk of lower respiratory tract infections in comparison to non-smoking women, even if breastfed [50]. This demonstrates that tobacco smoke during pregnancy may contribute to immunological dysfunction of the infant even after delivery.

### Conclusions

Exposure to xenobiotics of tobacco smoke by pregnant women:

1. Leads to an elevation of Cd concentration in the blood of smoking women. Cd negatively correlated with Zn and infant birth weight. The Zn/Cd ratio was lower in the blood of smoking women and correlated negatively with α1-globulins, α2-globulins and β-globulins, suggesting a potential influence of these metals on protein metabolism.
2. Leads to observable changes in the percentage of protein fractions, and declines in β-globulin and γ-globulin levels in the serum of smoking women in comparison with the control group.
3. May lead to the differences in levels of α1-anitrypsin and α2-macroglobulin among pregnant smoking and non-smoking women.

## Supporting Information

S1 Table Number of selected pregnancies, denials and exclusions. (DOCX)

S2 Table Number of selected pregnancies with diagnoses illnesses. (DOCX)
