# Supplementary material for: The Influence of Tobacco Smoke on Protein and Metal Levels in the Serum of Women during Pregnancy
Source: PLoS One. 2016 Aug 22;11(8):e0161342. doi: 10.1371/journal.pone.0161342 (PMC4993357; doi:10.1371/journal.pone.0161342)
Supplement: S1 Table — (DOCX) [file pone.0161342.s001.docx]

**Supplementary material**

**Table 1. Number of selected pregnancies, denials and exclusions.**

| **Total number of pregnant women involved into study: 70 pregnancies** | **The reason of exclusions:** |
| --- | --- |
|  | - 1 stillbirth with malformations |
|  | - 2 miscarriage before 15 gestational age |
|  | - 1 twins |
|  | - 11 women with diagnosed diabetes, insulin resistance, thyroid disease or autoimmune disease |
|  | **Total number of pregnant women after exclusions:  55 pregnancies** |
