# Supplementary material for: The Influence of Tobacco Smoke on Protein and Metal Levels in the Serum of Women during Pregnancy
Source: PLoS One. 2016 Aug 22;11(8):e0161342. doi: 10.1371/journal.pone.0161342 (PMC4993357; doi:10.1371/journal.pone.0161342)
Supplement: S2 Table — (DOCX) [file pone.0161342.s002.docx]

**Supplementary material**

**Table 2. Number of selected pregnancies with diagnoses illnesses**

| **Type of disorder** | **Number of pregnant women** |
| --- | --- |
| Thyroid disorders (Hashimoto thyroiditis, hyperthyroidism, hypothyroidism) | 3 |
| Insulin resistance | 6 |
| Gestational diabetes | 1 |
| Autoimmune disease (Systemic lupus erythematosus, antiphospholipid syndrome) | 1 |
